# Publisher Correction: Migration alters oscillatory dynamics and promotes survival in connected bacterial populations

**DOI:** 10.1038/s41467-019-08615-1

**Published:** 2019-02-12

**Authors:** Shreyas Gokhale, Arolyn Conwill, Tanvi Ranjan, Jeff Gore

**Affiliations:** 1 Physics of Living Systems, Department of Physics, Massachusetts Institute of Technology, Cambridge, MA 02139 USA; 2 John A. Paulson School of Engineering and Applied Sciences, Harvard University, Cambridge, MA 02138 USA

Correction to: *Nature Communications;* 10.1038/s41467-018-07703-y Published online 10 Dec 2018.

In the original version of this Article, an additional double-headed arrow was inadvertently included within Fig. 3e. This error has been corrected in both the PDF and HTML versions of the Article.

