# Supplementary material for: Migration alters oscillatory dynamics and promotes survival in connected bacterial populations
Source: Nat Commun. 2018 Dec 10;9:5273. doi: 10.1038/s41467-018-07703-y (PMC6288160; doi:10.1038/s41467-018-07703-y)
Supplement: Supplementary file 1 — Supplementary Information [file 41467_2018_7703_MOESM1_ESM.pdf]

Supplementary Information

Migration alters oscillatory dynamics and promotes survival in connected bacterial populations

Shreyas Gokhale*, Arolyn Conwill*, Tanvi Ranjan, and Jeff Gore

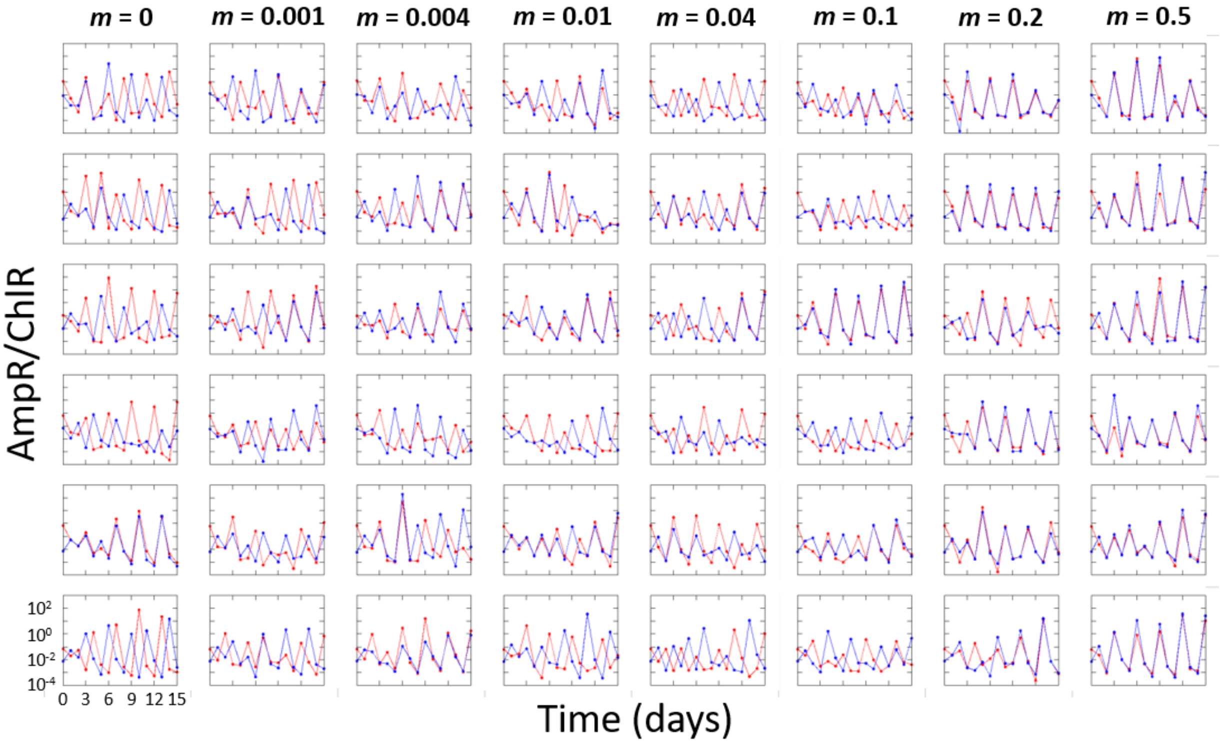

Supplementary Figure 1: Time series data for the ratio of AmpR to ChlR cells in two connected population patches in the benign environment (10 μg/ml of ampicillin, 8 μg/ml of chloramphenicol). Each column corresponds to a different migration rate, as shown, and each row corresponds to a different replicate. While some instances of in-phase synchronization are observed at low migration rates, all pairs are synchronized in-phase at $m = 0.2$ and $m = 0.5$. The scales on horizontal and vertical axes are shown at the bottom left.

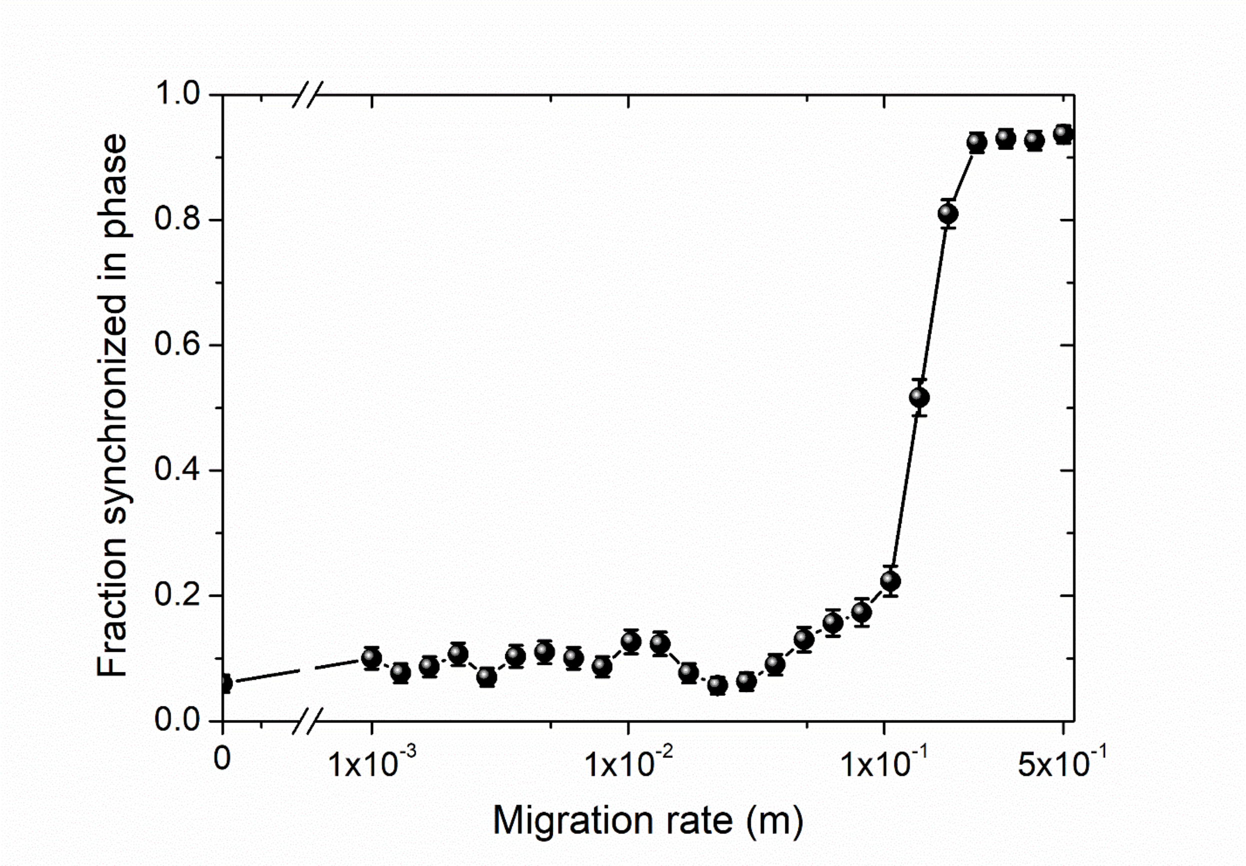

Supplementary Figure 2: Simulation data for the fraction of replicates for which the two coupled populations are synchronized in phase after 101 days, as a function of migration rate for the benign environment (10 μg/ml of ampicillin, 8 μg/ml of chloramphenicol). The simulations incorporate 15% noise in the migration and dilution steps of our simulations, to mimic the stochastic fluctuations resulting from our experimental protocol. The error bars are standard errors of proportion.

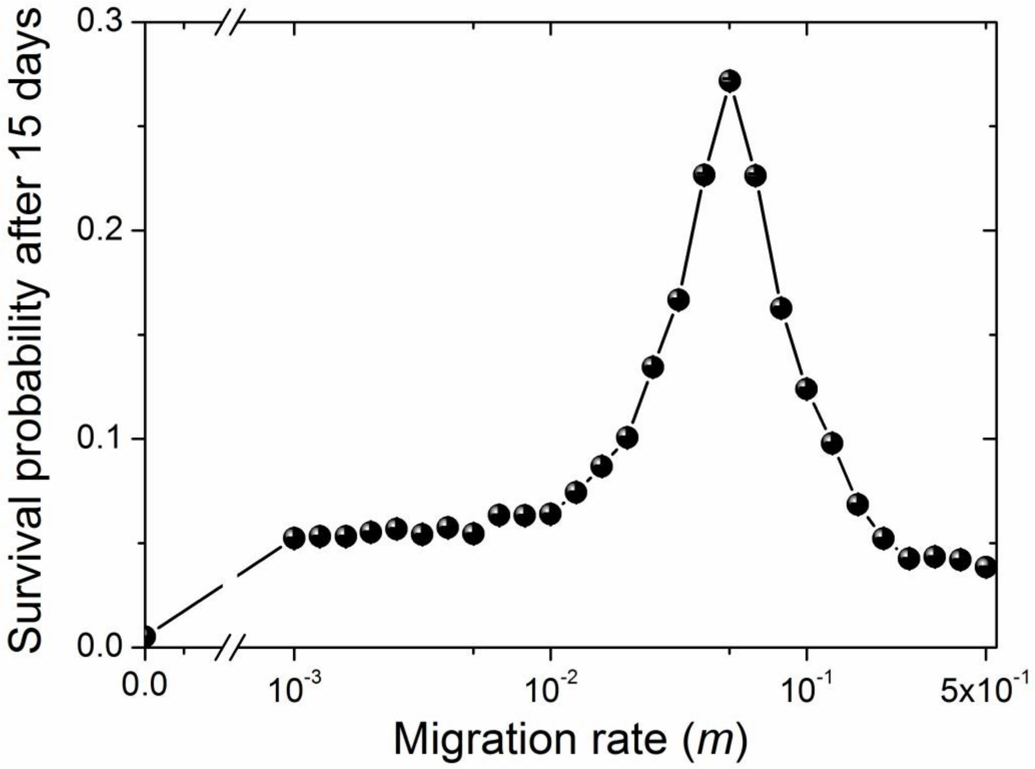

Supplementary Figure 3: Simulation data for survival probability after 15 days as a function of migration rate for the harsh environment (10 μg/ml of ampicillin, 16 μg/ml of chloramphenicol), showing a pronounced maximum near $m = 0.05$. The overall survival probability is smaller compared to that measured over 10 days (Fig. 5B in the main text), but the maximum is much sharper. Both the overall decrease in survival probability and the sharpness of the peak are consistent with the simulated distribution of survival times (Fig. 5A in the main text). The error bars, which are smaller than the symbol size, are standard errors of proportion.

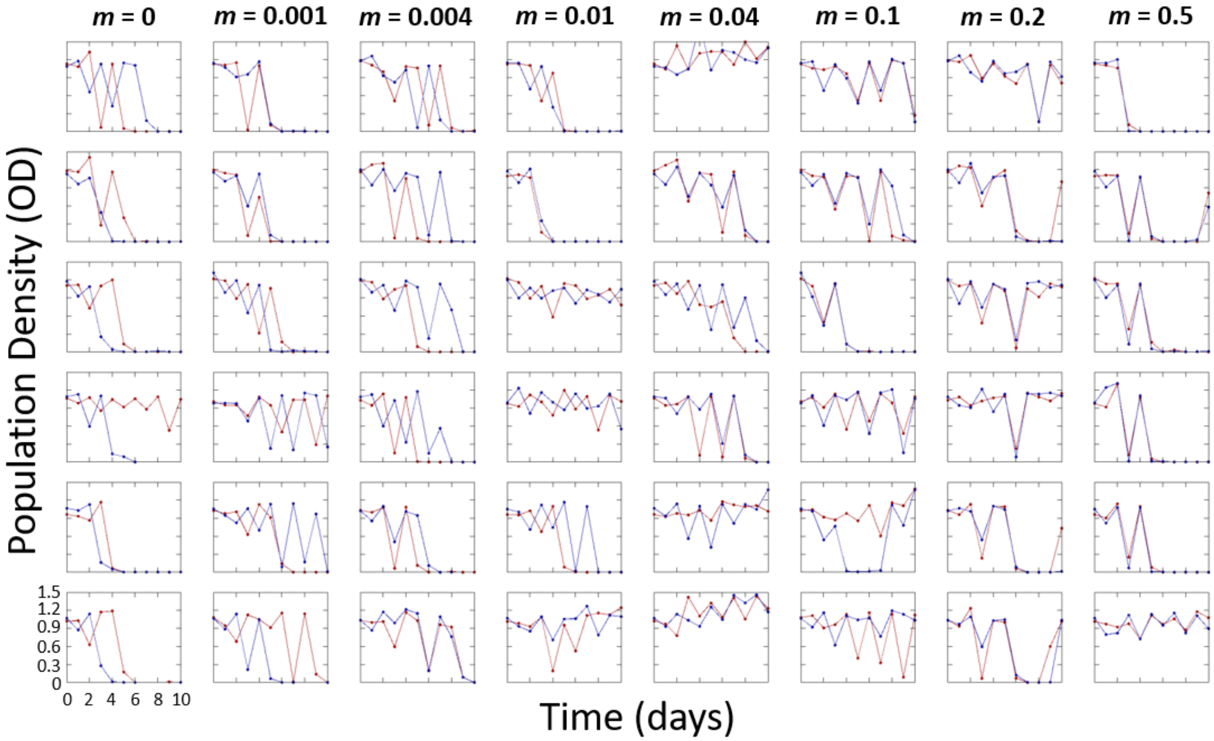

Supplementary Figure 4: Time series data for the population density, measured as the optical density (OD) at 600 nm, in the harsh environment (10 μg/ml of ampicillin, 16 μg/ml of chloramphenicol) for six replicates at each of all 8 migration rates studied. The fraction of surviving populations at $m = 0$ and $m = 0.5$ is noticeably smaller compared to that at intermediate migration rates. The scales on horizontal and vertical axes are shown on the bottom left.

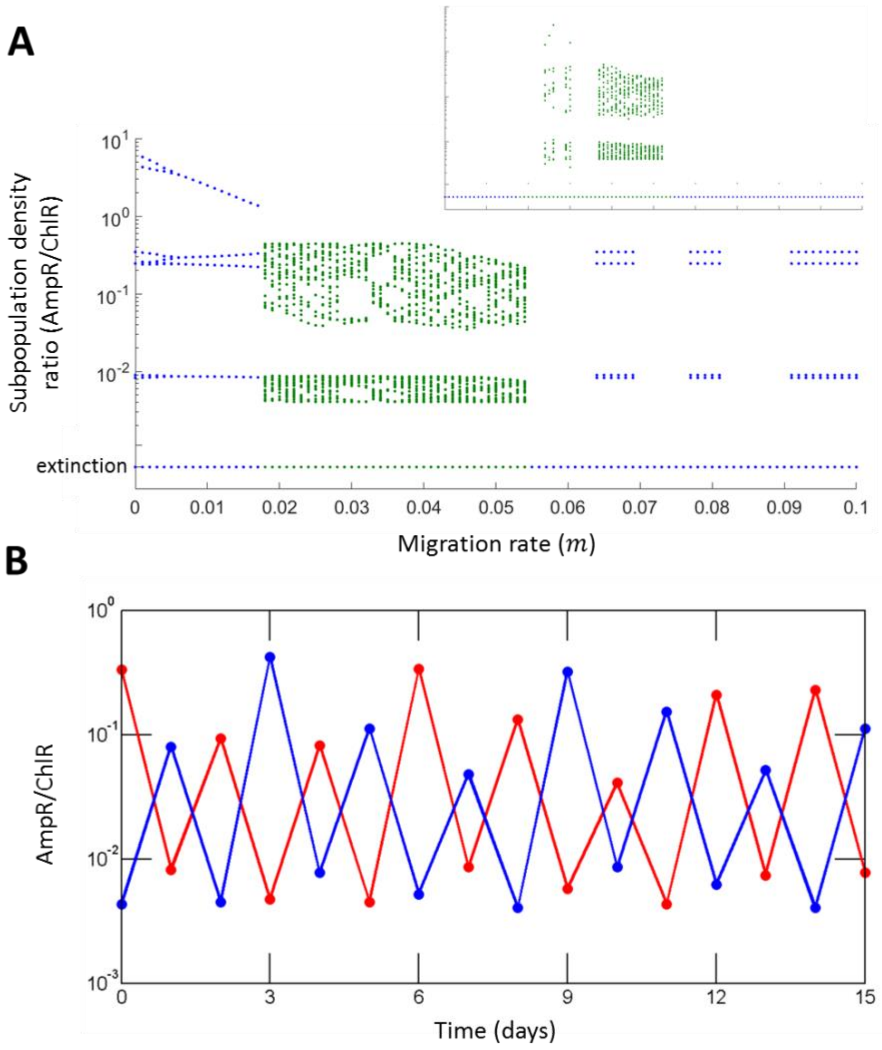

Supplementary Figure 5: Simulations reveal the presence of new dynamical states that appear over a narrow range of intermediate migration rates in the harsh environment (10 μg/ml of ampicillin, 16 μg/ml of chloramphenicol). **A)** Bifurcation diagram as a function of migration rate. Unique population values attained by patch A over the last 20 days of a deterministic simulation with 100 daily dilutions are plotted for each migration rate. The simulations were initialized at AmpR/ChlR ratios corresponding to the three phases of the period-3 limit cycle observed in benign conditions. Connected patches were initialized in different phases to minimize the probability of synchronization. Complex dynamics are observed over a narrow range of migration rates, whereas extinction dominates the high as well as low migration rate regimes. Oscillatory dynamics are observed in the low and high m regime as well. However, these dynamics are much more sensitive to noise than the ones observed at intermediate migration rates. In particular, the same bifurcation

diagram generated from simulations with 2% noise in migration rate and dilution factor (inset in **A**) shows that complex oscillatory dynamics at intermediate m are preserved over 100 daily dilutions whereas those at low as well as high m disappear. Axes in the inset are identical to those in (A). **B)** A representative simulation time series of the ratio of AmpR to ChlR cells at $m = 0.04$ showing complex deterministic oscillations that resemble noisy out of phase period-2 dynamics.

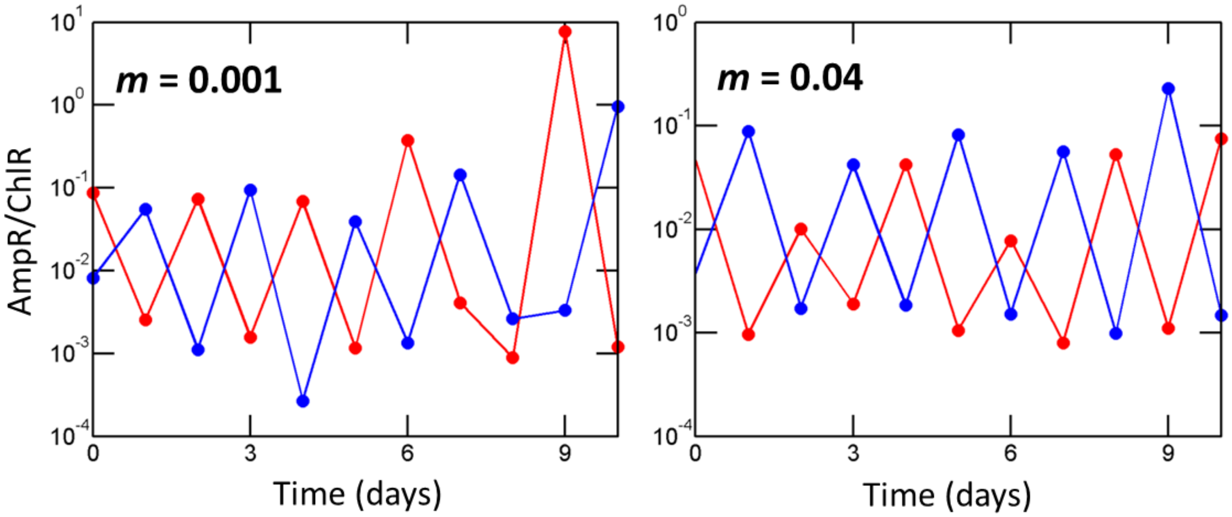

Supplementary Figure 6: Representative time series showing experimental evidence of out-of-phase period-2 oscillations in the harsh environment (10 μg/ml of ampicillin, 16 μg/ml of chloramphenicol) for $m = 0.001$ (left panel) and $m = 0.04$ (right panel). The period-2 oscillations at $m = 0.001$ appear to transform to a different period, illustrating that transient dynamics may also play an important role in governing survival in harsh environments.

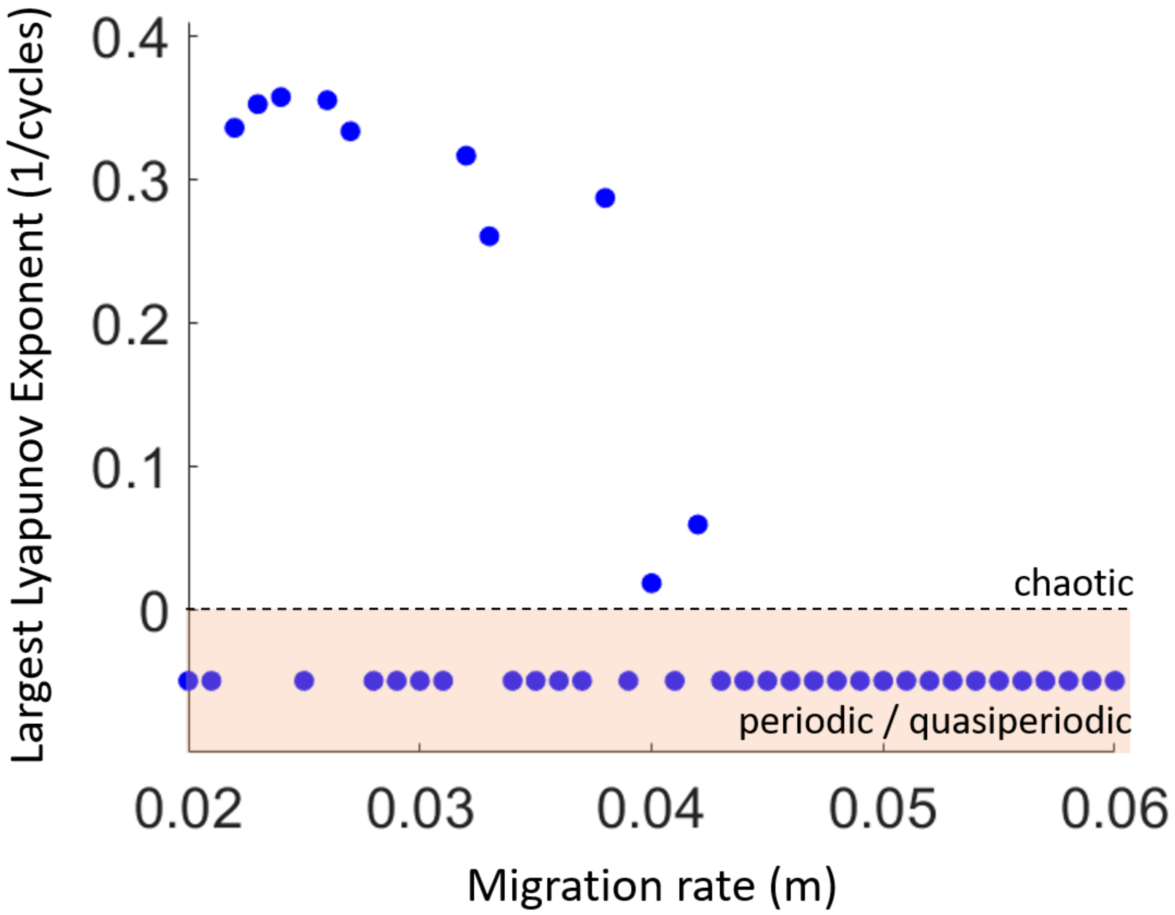

Supplementary Figure 7: Largest Lyapunov exponent as a function of migration rate for the deterministic mechanistic model in the harsh environment (10 μg/ml of ampicillin, 16 μg/ml of chloramphenicol). The algorithm only returns positive Lyapunov exponents, which indicate chaos. Points shown below 0 correspond to periodic or quasiperiodic dynamics. We computed the exponents from time series of the log ratio of AmpR cells to ChlR cells in a given patch. To minimize the effect of transients, time series were generated by simulating 10,000 daily growth-dilution-migration cycles.

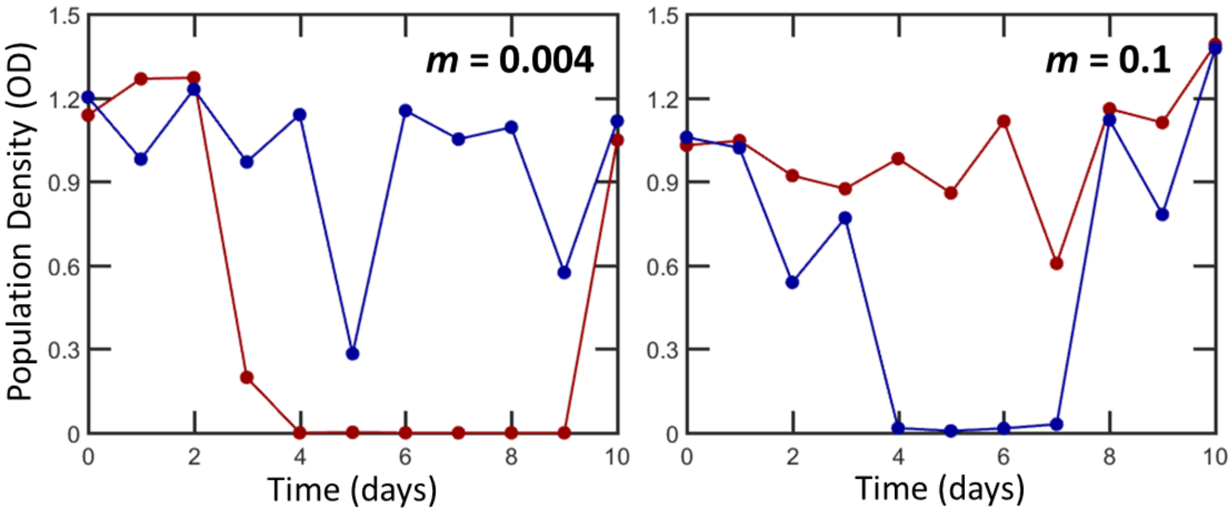

Supplementary Figure 8: Representative population density (OD) time series showing experimental evidence of re-colonization events. We observe that one of the populations appears to go extinct, but recovers a few days later due to migration from its partner.

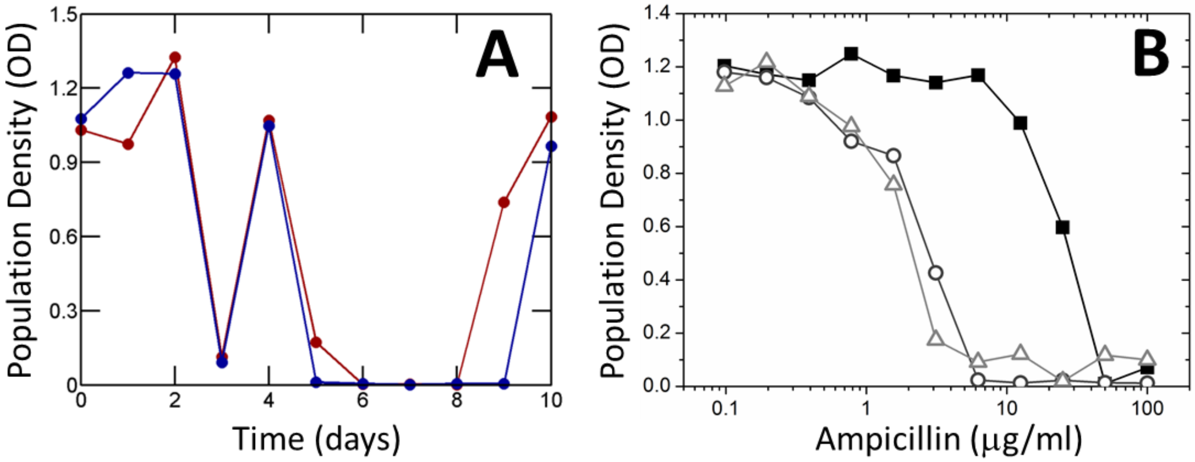

Supplementary Figure 9: Evidence for the chloramphenicol resistant strain (ChlR) evolving additional resistance to ampicillin in the harsh environment (10 μg/ml of ampicillin, 16 μg/ml of chloramphenicol). **A)** A representative population density (OD) time series ($m = 0.5$) in which both populations in a pair appear to go extinct, but one of them (red curve) rises again on day 9 and re-colonizes its partner, as seen from the rise in the blue curve on day 10. **B)** We measured the concentrations of ampicillin that inhibit growth of the ancestral ChlR strain before the migration experiment (open grey triangles), a biological replicate of the same ancestral ChlR strain after the experiment (open dark grey circles), and the evolved ChlR strain at the end of 9 days of the migration experiment (solid black squares). We observe that the evolved strain has tenfold higher resistance to ampicillin as compared to the ancestral strain.

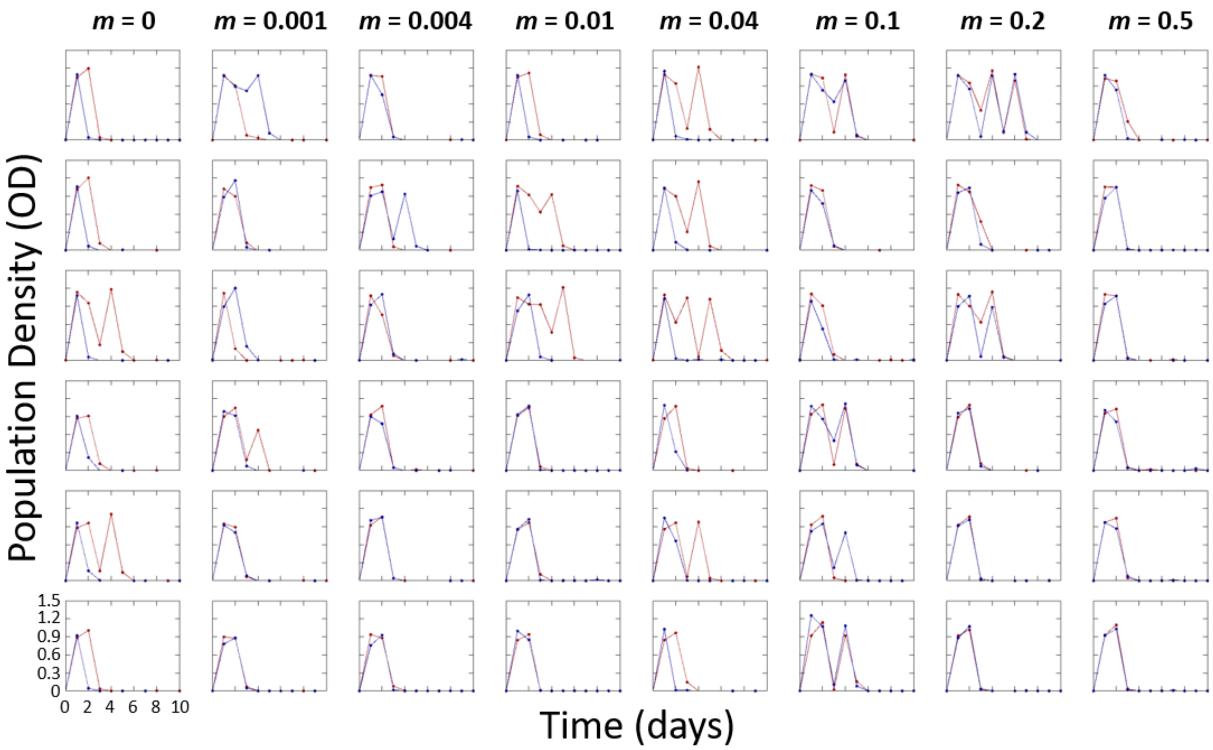

Supplementary Figure 10: Time series data for the population density (OD) in an extremely harsh environment (10 μg/ml of ampicillin, 20 μg/ml of chloramphenicol) for all 8 migration rates studied. All populations become extinct within 7 days and there is no evidence for evolution of higher antibiotic resistance.

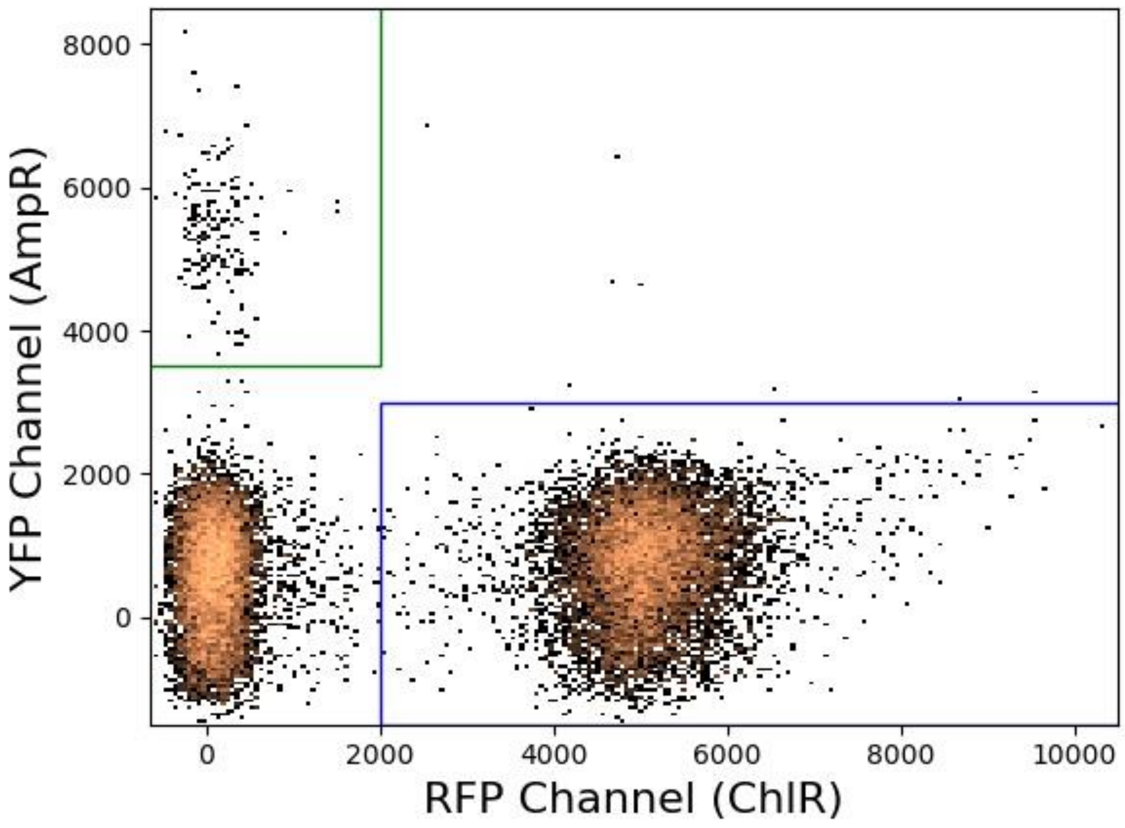

Supplementary Figure 11: Pseudocolor plot exemplifying our flow cytometry gating strategy, for a representative data set in the benign environment (10 μg/ml of ampicillin, 8 μg/ml of chloramphenicol, $m = 0.004$). We set the detection threshold to be low, such that the detector noise exhibits a well defined peak at low intensities in both RFP and YFP channels (bottom left peak in the pseudocolor plot). It is evident that the fluorescence peaks for AmpR cells (YFP channel) as well as ChlR cells (RFP channel) are clearly distinguishable from noise. We plotted histograms of fluorescence intensities for RFP as well as YFP channels and chose the intensity values corresponding to the histogram minimum as gating thresholds. Our results are unaffected by small changes in gating thresholds.

Parameter	Meaning	Value used
γ_1^R	Growth rate of AmpR cells in the absence of chloramphenicol	1.18/hr
γ_2^R	Growth rate of ChlR cells in the absence of ampicillin	1.21/hr
γ_2^D	Death rate of ChlR cells at high ampicillin concentrations	0.25/hr
I_{12}	Inhibitory concentration for AmpR cells in chloramphenicol	0.65 μg/ml
I_{21}	Inhibitory concentration for ChlR cells in ampicillin	1.3 μg/ml
K_m	Michaelis-Menten constant for ampicillin inactivation	12 μg/ml
V_{max}	Maximal hydrolysis rate of ampicillin	20000 $\frac{\mu g}{ml.hr.K}$
c_2	Inactivation rate of chloramphenicol	12.8 / (hr.K)
t_{lag}	Lag time during which no growth occurs but antibiotics can be deactivated	1 hr
K	Carrying capacity (density)	1
N_{min}	Lowest viable population density (finiteness of population size)	1 cell ≡ 10^{-8}

Supplementary Table 1: The table lists the values of parameters used in our simulations. The population density is measured in units of the carrying capacity. In experiments, the carrying capacity of ChlR and AmpR cells is ~2.3 x 10^6 cells/μl. All parameter values except the one used for c_2 are identical to those used in our previous work (Ref. 31 in the main manuscript). We chose the value of c_2 to be about 15% lower than that used previously so that the survival probability in the absence of migration was close to the value measured in experiments.
